# Oxidative stress and cellular pathologies in Parkinson’s disease

**DOI:** 10.1186/s13041-017-0340-9

**Published:** 2017-11-28

**Authors:** Lesly Puspita, Sun Young Chung, Jae-won Shim

**Affiliations:** 1 Soonchunhyang Institute of Medi-bio Science (SIMS), Soonchunhyang University, 25, Bongjeong-ro, Dongnam-gu, Cheonan-si, 31151 South Korea; 2 Center for Stem Cell Biology, Sloan-Kettering Institute, New York, NY 10065 USA

**Keywords:** Alpha-synuclein, Dopamine neurons, Mitochondria, Oxidative stress, Parkinson’s disease, Reactive oxygen species, Unfolded protein response

## Abstract

Parkinson’s disease (PD) is a chronic and progressive neurodegeneration of dopamine neurons in the substantia nigra. The reason for the death of these neurons is unclear; however, studies have demonstrated the potential involvement of mitochondria, endoplasmic reticulum, α-synuclein or dopamine levels in contributing to cellular oxidative stress as well as PD symptoms. Even though those papers had separately described the individual roles of each element leading to neurodegeneration, recent publications suggest that neurodegeneration is the product of various cellular interactions. This review discusses the role of oxidative stress in mediating separate pathological events that together, ultimately result in cell death in PD. Understanding the multi-faceted relationships between these events, with oxidative stress as a common denominator underlying these processes, is needed for developing better therapeutic strategies.

## Introduction

Parkinson’s disease (PD) is the second most common neurodegenerative disorder, characterized by serious movement disturbances such as tremor, rigidity, and bradykinesia. It is a chronic condition attributed by the selective degeneration of dopamine (DA) neurons in the midbrain substantia nigra (SN). By the time patients experience these symptoms, a portion of DA neurons that project from the SN to the striatum have already degenerated [1, 2]. Appearance of insoluble inclusions in neurons called Lewy bodies, which mainly consist of α-synuclein, is a major hallmark of this disease [1]. Based on its progressive nature, it is unlikely that the disease pathogenesis is triggered by an acute toxicity that kills cells directly. Instead, it is possible that an ongoing process such as oxidation is responsible for the gradual dysfunction that manifests across myriad cellular pathways throughout the disease trajectory.

Most PD cases are sporadic, with only about 10% associated with an inherited genetic component. Even though familial cases comprise only a minor subset of the overall PD pool, examining PD-related monogenic mutations is a valuable method of understanding disease pathogenesis and cell death which may have implications for studying the disease at large. PTEN induced putative kinase 1 (PINK1), Parkin, DJ-1, leucine-rich repeat kinase 2 (LRRK2) and α-synuclein are among the proteins which have been strongly linked to the familial forms of PD [3–7]. Of these, α-synuclein is most commonly associated with PD pathogenesis for its predominance in Lewy bodies, which develop and aggregate throughout disease progression [8, 9]. PINK1 and Parkin are involved in mitochondria-related autophagy, whereas the loss of function of these proteins leads to the accumulation of damaged mitochondria [10, 11]. DJ-1 is involved in a wide range of cellular functions, two of which include its roles as a sensor for oxidative stress and as a redox-chaperone protein [12, 13]. The physiological function of LRRK2 is less understood but neurons with mutations in this protein exhibit greater vulnerability to mitochondrial toxins [14].

Reactive oxygen species (ROS) are normally produced in the cell during mitochondrial electron transfer chain (ETC) or redox reactions and are in fact a necessary component of cellular homeostasis. As an example, several enzymes in mitogen-activated protein kinase and phosphoinositide 3-kinase pathways, which are pivotal in mediating cellular responses to growth hormones and cytokines, are regulated directly by ROS [15–17]. Yet despite the importance of ROS in normal physiology, antioxidant proteins like superoxide dismutase (SOD) and glutathione (GSH) also prevent ROS levels from getting too high [18]. Failure of these antioxidants in regulating ROS levels leads to oxidative stress, which can have a variety of detrimental effects. Random oxidation of macromolecules inside the cell can damage cellular structures and even lead to cell death [19–21]. Previous publications have reported evidence of oxidative stress through the detection of oxidized DNA, lipids, and proteins in the brain tissues of both familial and sporadic PD patients [22, 23]. Since oxidative stress increases the chance of spontaneous mutations, it is possible that this can trigger mutations that make cells more vulnerable to dysfunction. Interestingly, in the SN of healthy individuals, the concentration of oxidized proteins was found to be twice that of the caudate, putamen, and the frontal cortex, indicating that susceptibility of SN to oxidative stress may contribute to the selective neuronal degeneration [24].

While many studies in the past have examined dysfunctional cellular processes in PD independently of each other, in our recent study, we sought to develop a more comprehensive understanding of the disease by examining how those processes might be interconnected [25]. Using induced pluripotent stem cell (iPSC)-derived midbrain DA neurons from patients with PINK1 or Parkin mutations, we first noted the presence of abnormal mitochondria. We also observed cytosolic α-synuclein and DA accumulation, with increased sensitivity toward oxidative stress-inducing agents, in the mutant lines [25]. Similarly, another group which had utilized LRRK2 mutant iPSC-derived neurons noted elevated expression of genes involved in oxidative stress regulation, and α-synuclein levels. Moreover, they observed cells’ increased vulnerability towards H_2_O_2_, 6-hydroxydopamine (6-OHDA), and MG132, a proteasome inhibitor [26]. Together, these results reflect the idea that a single mutation can profoundly disrupt cellular homeostasis, implying that PD progression may be the result of a multitude of interactions between the various pathogenic phenotypes linked to cellular stress.

**Fig. 1:**
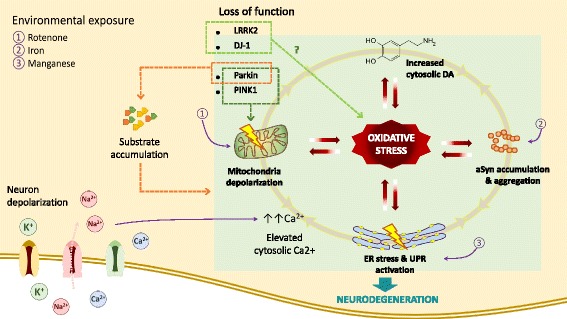
Central role of chronic oxidative stress in mediating PD progression. Mitochondria depolarization, ER stress, α-synuclein accumulation and increased level of cytosolic DA are known PD phenotypes that potentially contribute to cellular oxidative stress, alone or by interacting with each other. ER regulates cytosolic calcium and thus prevents excess uptake by MCU that otherwise can stimulate ETC and ROS production in mitochondria, an essential function in neurons with intense depolarization. α-synuclein accumulation can contribute to ER stress by binding to ER chaperones and disturbing vesicle trafficking between ER and Golgi. While UPR activation also stimulate α-synuclein aggregation by unclear mechanism. Utilization of DA risks midbrain DA neurons to oxidative damage by DA metabolism. Additionally, DA has been shown to trigger α-synuclein oligomer formation and mitochondria depolarization. Altogether, these phenotypes produce oxidative environment that further amplifies the damage. Mutations that disturb the function of LRRK2, DJ-1, Parkin and PINK1 have been linked to familial cases of PD. Parkin is an E3 ubiquitin ligase and dysfunctionality of this protein results in accumulation of its substrates. Together with PINK1, Parkin is also responsible to clear up damaged mitochondria. One of DJ-1 putative role as sensor of oxidative stress may be necessary in cell protection. Single mutation in *LRRK2* resulted in increased susceptibility towards oxidative stressor, even though the mechanism is less understood. Pesticide rotenone, iron and manganese also cause cellular oxidative stress by triggering mitochondria depolarization, α-synuclein oligomerization together with ROS production and UPR activation, respectively. Long term exposure of those substances has been linked with higher risk of developing sporadic PD

While oxidative stress has been thoroughly researched, advances in stem cell technology have engendered a wide range of tools with which to study and model diseases in vitro. As demonstrated by our study as well as many others, iPSCs have made it possible to study specific disease mutations using patient derived cells, which has been especially valuable in modeling neurodegenerative diseases which lack authentic animal models. Moreover, because PD is diagnosed only when the degeneration of midbrain DA neurons has already progressed considerably, neurons from PD patient-derived iPSCs enable researchers to carefully track even minor disturbances that precede major pathogenic processes. Based on our iPSC-based findings demonstrating the contribution of oxidative stress toward triggering dysfunctional processes, this review explores how oxidative stress may play a central role in mediating disease progression (Fig. 1).

### The mitochondrion is a key site of ROS production and a target of ROS-induced damage

In the inner membrane of mitochondria, electrons are transferred through a series of protein complexes via redox reactions to oxygen, the last electron acceptor. As the electrons pass, some protons are translocated by the electron carriers from the matrix to the mitochondrial intermembrane space, thereby creating a proton gradient. Protons flow back into the mitochondrial matrix following its gradient, concurrently providing energy for the ATP synthase to phosphorylate ADP into ATP. This entire process, which is a critical means of energy production, produces ROS as a major byproduct [27]. Premature electron leakage in ETC Complex I (Nicotinamide adenine dinucleotide [NADH] dehydrogenase) and Complex III (cytochrome bc1) to oxygen, is the main source of mitochondrial superoxide anions (O_2_^−^) [28, 29]. Production of ROS from the mitochondrial action is physiologic, but dysfunction of ETC in damaged mitochondrial causes excessive ROS production, which is quite detrimental to cells.

The involvement of mitochondria in PD pathogenesis was first brought to light after individuals consumed illicit drugs contaminated with 1-methyl-4-phenyl-1,2,3,6-tetrabydropyridine (MPTP). Symptoms resembling those present in PD were observed soon after drug intake with postmortem analyses revealing destruction of the SN [30]. Subsequent studies explained that 1-methyl-4-phenylpyridinium (MPP+), the toxic bioactive form of MPTP, undergoes oxidation by monoamine oxidase B (MAO-B) and enters the DA-producing neurons in the SN via the DA reuptake system [31]. Upon entering the cell, MPP+ inhibits the mitochondrial ETC Complex I enzyme, and NADH-ubiquinone oxidoreductase (EC 1.6.5.3), resulting in electron leakage and ROS generation in mitochondria [32]. Similarly, rotenone, a pesticide, also induces parkinsonism by inhibiting ETC Complex I. Due to its hydrophobicity, rotenone easily crosses biological membranes independently of the DA transporter and its delivery causes systemic inhibition of the mitochondrial ETC. Notably, the degeneration is specific to midbrain DA neurons, while other DA-producing neurons in the ventral tegmental area (VTA) may be relatively spared [33]. Such decline of Complex I activity and elevated intracellular ROS have been verified in the SN of the post-mortem brain of PD patients [34, 35].

Further supporting the importance of the mitochondria and its relevance in PD is the fact that PD-related genes such as *PINK1*, *PARK2* (Parkin), *DJ-1* and *LRRK2* encode proteins that regulate mitochondrial and ROS homeostasis [3, 4, 6, 7, 36]. PINK1 is a mitochondrial protein that is degraded rapidly in healthy mitochondria. In defective mitochondria, which may exhibit high levels of oxidative stress, decreased membrane potential, or the presence of misfolded proteins, the degradation of PINK1 is impeded, leading to the accumulation of PINK1 on the mitochondrial outer membrane. PINK1, which phosphorylates Parkin at Ser65, induces E3 ubiquitin ligase activity of the enzyme and its recruitment to the mitochondria. Parkin modifies proteins on the mitochondrial membrane by adding ubiquitin chains that function as signals for autophagy. The mitochondria-specific autophagy process, also known as mitophagy, ultimately results in mitochondria engulfment and degradation [10, 11] which has been demonstrated experimentally where the systemic knockout of Parkin in mice resulted in elevated intracellular ROS levels in the VTA and a reduction in proteins involved in ETC and oxidative stress regulation [37]. In drosophila, PINK1 null mutants exhibited reduced mitochondrial membrane potential, suboptimal ETC activity, as well as a reduction in synaptic neurotransmitter release in neural cells [38]. Moreover, accumulation of damaged mitochondria in neuronal axons, as observed in PINK1 knock-out mutants, could be a source of ROS and oxidative damage [39].

Defects in mitophagy and increased oxidative stress might also partially explain the specificity of the phenotypes in DA neurons. As shown in PINK1 null mutants that display reduced neurotransmitter release, mitochondria are critical in cells with actively firing axons [38]. In an unusual case of a woman with a homozygote recessive Parkin mutation, she remained free of PD symptoms even through her eighth decade, while her relative carrying the same mutation exhibited early onset of PD. When comparing their fibroblasts, it was found that Nip3-like protein X, which may mediate a Parkin/PINK1-independent pathway in eliminating damaged mitochondria, was highly upregulated in the asymptomatic carrier. Levels of mitochondrial membrane potential, oxygen consumption rate, and resistance capacity toward protonophore carbonyl cyanide-m-chlorophenylhydrazone (CCCP) were higher in fibroblasts derived from the asymptomatic carrier than those of the individual with PD symptoms. Although the study was conducted in fibroblasts and not midbrain DA neurons, the data strongly suggested that failure in mitochondria clearance or in other words, accumulation of defective mitochondria, was an important factor in mediating PD pathology [40]. Another protein related to a recessive form of PD, DJ-1, contains a cysteine residue (C106) that is vulnerable to oxidation during oxidative stress. Oxidation of C106 leading to the formation of cysteine-sulfinic acid has been verified using mass spectrophotometry [41] and crystal analysis of DJ-1. During oxidative stress, the oxidized DJ-1 may translocate to the outer membrane of mitochondria and it has been shown to prevent MPP + −induced cell death, the mechanism of which remains unclear [12]. In line with this finding, homozygous mutation of DJ-1 has been linked to increased mitochondrial oxidative stress in human iPSC-derived DA neurons, a feature that was accompanied by accumulation of α-synuclein and oxidized form of DA [42].

As one of the main sites of ROS production, mitochondria are particularly susceptible to oxidative stress-induced damage. Unlike nuclear DNA, mitochondrial DNA (mtDNA) are unprotected by histone proteins and therefore are easy targets of oxidation [43]. ROS production and mtDNA damage has been shown to increase with age, up to 10–20 folds higher than in nuclear DNA [44, 45]. Since most of the proteins coded by mtDNA are involved in ETC, mutations and deletions in mtDNA would likely disturb ETC and increase ROS formation, creating a vicious cycle further inflicting mitochondrial damage [46]. Another mechanism of how this cycle might work involves nitrosative stress induced by either mitochondrial toxins or mutated α-synuclein proteins. In Ryan et al., these were shown to cause sulfonation on myocyte-specific enhancer factor 2C (MEF2C). This modification inhibits MEF2C transcriptional activity and consequently, decreases expression of the target genes. One of the important genes regulated by MEF2C encodes peroxisome proliferator-activated receptor gamma coactivator 1-alpha (PGC-1a), a master regulator of mitochondria biogenesis. Therefore, failure to express PGC-1a implies dysfunction among mitochondria [47].

### ER protein folding and calcium storage functions are prominent sources of ROS

The endoplasmic reticulum (ER) is the site of secretory protein production and post-translational modifications such as protein folding and glycosylation. Protein folding is a process that is greatly affected by the redox status of the ER lumen as the formation of disulfide bonds requires a highly oxidizing environment. During disulfide bond formation, electrons are transferred from the target protein to oxygen by protein disulfide isomerase and ER oxidoreductin-1, which forms ROS as byproduct [48, 49]. Quantitative analyses of protein synthesis and processing suggest that disulfide bond formation produces approximately 25% of total ROS in the ER lumen [50].

Another vital role of the ER entails regulation of intracellular calcium, involving the release or absorption of calcium to regulate its cytoplasmic concentration levels. Failure to maintain the calcium concentration in homeostasis and accumulation of misfolded proteins may lead to the activation of the unfolded protein response (UPR) [51]. UPR is a protective mechanism which is initiated by three proteins in the ER membrane: inositol-requiring enzyme 1 (IRE1), activating transcription factor (ATF) 6, and pancreatic ER kinase (PKR)-like ER kinase (PERK) which each bind to GRP78/BIP (binding immunoglobulin protein), a chaperone in the ER lumen. In the presence of misfolded proteins, BIP dissociates from the membrane proteins to bind the misfolded proteins. Dissociation from BIP activates the three membrane proteins and the following pathways. IRE1 splices the intron in X-box binding protein 1 (XBP1) RNA to produce its translationally active form. After dissociation from BIP, ATF6 is translocated from the ER to the Golgi apparatus, where it is cleaved and activated. Both XBP1 and ATF6 act as transcriptional regulators of ER chaperones and ER-associated degradation pathways, which are essential for reducing ER stress and promoting cell survival. Lastly, BIP dissociation triggers autophosphorylation of PERK into phosphorylated PERK (pPERK). Phosphorylation of initiation factor 1 subunit α (eIF2α) by p-PERK results in global attenuation of protein translation. However, the attenuation does not apply to certain PERK-downstream proteins like ATF4. Prolonged expression of ATF4 can trigger the expression of another transcription factor, C/EBP homologous protein (CHOP), and the downstream apoptotic pathway [52, 53]. Activation of the ATF4/CHOP pathway could lead to apoptosis and has thus far been suggested as a part of the neuronal apoptotic signaling pathway [54].

The ER’s function in regulating calcium and the downstream events can greatly affect mitochondria. Calcium leakage from the ER into the cytosol due to ER stress can trigger excessive calcium intake by the mitochondria via mitochondrial calcium uniporter (MCU) [55–58]. ER-to-mitochondria transfer of calcium is facilitated by mitochondrial-associated membrane (MAM) which brings both organelles in close proximity [59, 60]. As visualized in human fibroblasts using green fluorescence protein-tagged ER membrane protein and the mitochondrial dye tetramethylrhodamine methyl ester, enhanced proximity of both organelles was observed in fibroblasts containing Parkin mutation compared to those of control fibroblasts [61]. As expected, calcium transfer to mitochondria was equally increased. Downregulation of Parkin substrate mitofusin 2, another MAM tethering protein [62], or exogenous expression of Parkin was shown to rescue the disturbance of calcium homeostasis in the mutant fibroblasts [61]. Moreover, it has been found that increased calcium in the mitochondria leads to the stimulation of ETC and can exacerbate ROS formation, or induce the activation of mitochondria-related apoptotic pathways [63, 64].

In PD cases, immunoreactivity of pPerk and peIF2a were observed by Hoozemans and colleagues in the SN of patients compared to those of healthy individuals [65]. Another study revealed that treatment with either MPP+ or 6-OHDA induced the changing of UPR proteins such as expression of BIP and CHOP, and phosphorylation of PERK and eIF2α, while relieving ER stress with salubrinal, a selective inhibitor of eIF2α dephosphorylation, attenuated mitochondrial toxin-induced cell death [66]. ER stress might also impact cellular oxidative stress through regulation of mitochondrial clearance. Putative ATF4 binding sites were found upstream of the transcriptional start site and in the first intron of the human *PARK2* gene. To verify this, ER stress was triggered in SH-SY5Y, human embryonic kidney T293 cells, and mouse embryonic fibroblasts by using the ER Ca2 + −ATPase inhibitor thapsigargin, the N-glycosylation inhibitor tunicamycin, and inducing amino acid starvation. In all experiments, upregulation of Parkin mRNA was observed. Data from a luciferase reporter assay and chromatin immunoprecipitation provided further evidence that ATF4 functions as a transcriptional regulator of Parkin [67]. A separate study also supported this idea by showing that ATF4 protects from neuronal apoptosis by regulating the level of Parkin [68]. Altogether the studies suggest the failure in degradation of Parkin’s substrates that is required during UPR activation may contribute to the neurodegeneration in PD with Parkin mutation. Nonetheless, further research is needed to determine whether ER stress is directly involved in the pathogenic mechanism of Parkin-associated PD.

Increased risk of developing sporadic PD after overexposure to manganese, copper, iron and mercury has been studied for decades [69, 70] where it has been suggested that α-synuclein, ER stress, and oxidative stress are involved in manganese toxicity in neurons. After 24 h of manganese treatment, α-synuclein oligomerization, elevated ROS, and oxidative damage of macromolecules have been observed in primary neuronal cultures. As an example, pre-treatment with GSH partially rescued the α-synuclein oligomerization and neuronal damage while H_2_O_2_ accelerated the process [66]. Xu et al. demonstrated that manganese treatment on rat brain slices induced expression of UPR proteins and apoptotic cell death [71]. In the rat models where α-synuclein expression was silenced by siRNA, apoptosis by manganese treatment was less pronounced. Furthermore, pPERK, pEIF2a, and ATF4 protein levels were also lower than those of wild type (WT) rats, despite the absence of changes in pIRE1 and sXBP1, suggesting α-synuclein involvement in the UPR PERK pathway [71]. In mice with mutant A53T, interaction between α-synuclein with ER chaperones in ER lumen was observed, indicating that abnormalities in α-synuclein alone could trigger ER stress and the downstream response [72]. Activation of IRE1α/XBP1 axis of UPR was also found in iPSC-derived DA neurons obtained from patients with α-synuclein triplication. In support of this, postmortem analyses conducted in the same study verified the presence of pIRE1 together with elevated level of α-synuclein in the brain [73]. Additionally, a recent study with an animal model reported that tunicamycin, an ER stress inducer also affected the aggregation process of α-synuclein [74].

### Alpha-synuclein is affected by and contributes to oxidative stress, by binding with iron and mitochondrial membrane proteins

Alpha-synuclein is a 140 kDA protein encoded by the *SNCA* gene. As the main component of Lewy bodies, α-synuclein is a well-known player in PD pathogenesis as duplication, triplication, and point mutations in its N-terminal region (A30P, A53T and E46K) are connected to familial PD [8, 9]. A growing body of work suggests that the monomer and tetramer types are the physiological forms of α-synuclein, while oligomers and fibrils are the pathogenic forms [75, 76]. Abundance of fibril α-synuclein was detected in Lewy bodies in several studies; however, abnormal accumulation of the soluble monomer form that leads to formation of oligomers and fibrils has also been proposed as a key pathogenic event in the early stages of PD [9, 77].

Spontaneous oligomerization and fibrilization of α-synuclein have also been observed in vitro, with mutated α-synuclein oligomerizing faster than the WT form of the protein [78]. In the same study, DA treatment increased the rate of polymerization in both mutated and WT forms of α-synuclein. Evidence of α-synuclein accumulation as a signature of disease initiation was shown in Nurr1+/tyrosine hydroxylase (TH) + neurons derived from a patient’s iPSCs. Additionally, α-synuclein accumulation was observed in neurons derived from patients with PINK1 or Parkin mutations, along with abnormal mitochondrial morphology and increased sensitivity towards oxidative stress [25]. Deas et al. suggested that interactions between α-synuclein oligomers and metal ions may induce oxidative stress in human iPSC-derived neurons. Neurons with α-synuclein triplication were reported to have a higher basal level of oxidative stress. When monomer, oligomer, or fibril forms of exogenous α-synuclein were added, α-synuclein oligomers triggered oxidative stress more potently than monomers and fibrils. Neurons treated only with oligomer α-synuclein demonstrated a reduction in the level of GSH and an increase in lipid peroxidation [79]. The propensity of oligomers to induce ROS production was significantly reduced in the presence of metal chelators such as deferoxamine, indicating that α-synuclein oligomers produce superoxide radicals by binding to transition metal ions such as copper and iron [80]. In vitro incubation of α-synuclein with iron resulted in the formation of H_2_O_2_ and hydroxyl radicals, a finding that supported iron-rich SN neurons’ selective vulnerability toward oxidative stress [81, 82]. Increased iron has also been detected in the SN of postmortem PD brains as well as living PD patients using magnetic resonance imaging [83–85]. Greater colocalization of iron and DA was found in the SN compared to those in the VTA [86], and given the ability of both substances to modify α-synuclein [81, 82], this can explain the selective vulnerability of this region.

Oxidative stress upon accumulation of α-synuclein can also be mediated by direct interaction between α-synuclein and mitochondrial membrane protein. In fact, α-synuclein has been demonstrated to disturb the translocation of nuclear-encoded mitochondrial proteins into the mitochondria by binding to translocase of the outer membrane (TOM)20 in both rotenone-treated and human α-synuclein-overexpressing animal models. The binding prevents the interaction of TOM20 with the co-receptor TOM22 and the subsequent translocation of mitochondrial-targeted proteins, which include some subunits of Complex I. As a result, mitochondrial ETC is rendered defective and intracellular oxidative stress escalates [87].

While α-synuclein toxicity can contribute in elevating cellular oxidative stress, it has also been suggested that oxidative stress can trigger the toxicity of α-synuclein. One consequence of chronic oxidative stress is lipid peroxidation of polyunsaturated fatty acids in the cell membrane, as observed in post-mortem SN [88]. A product of lipid peroxidation, 4-hyroxy-2-nonenal, prevents fibrillation of α-synuclein and supports the formation of secondary beta sheets and toxic soluble oligomers in a dose-dependent manner [89, 90]. Thus, oxidative stress can also influence α-synuclein toxicity and mediate PD pathogenesis. Incubation of α-synuclein monomers with cytochrome c/H_2_O_2_ led to α-synuclein aggregation in vitro by catalyzing the crosslinking of α-synuclein tyrosine residues through 3,3′-dityrosine bond formations [91, 92]. Colocalization of cytochrome c and α-synuclein has also been detected in the Lewy body of PD brains [91].

Since accumulation of α-synuclein monomers initiates the formation of aggregates, some studies have shifted their focus toward the α-synuclein degradation process. Both ubiquitin-proteasome system (UPS) and autophagy-lysosomal pathway have been linked with α-synuclein degradation [93], with more recent publications suggesting that UPS is the main α-synuclein degradation pathway under normal physiological conditions, while the lysosomal pathway is more responsive to stress or when α-synuclein is overly abundant [94, 95]. Chaperone-mediated autophagy, a subtype of lysosomal pathway, works with the help of cytosolic chaperone Hsc70 that recognizes the KFERQ peptide motif in α-synuclein [78, 95]. Then α-synuclein is delivered to a receptor in the lysosomal membrane and translocated into the lysosome, where enzymatic degradation takes place. However, in a highly oxidizing environment, the oxidized form of α-synuclein cannot be efficiently degraded, resulting in its accumulation and aggregation [78]. Its accumulation also affects vesicle trafficking between the ER and the Golgi, and reduces lysosomal degradation capacity [96–98]. Supporting this, α-synuclein had been shown to inhibit ER-Golgi transit of COPII vesicles which carries ATF6 to its activation site at the Golgi apparatus. Consequently, the protective ATF6 pathway of UPR signaling was blocked [99].

Oxidative stress and α-synuclein accumulation preceding diminished lysosomal proteolysis has also been observed in patient-derived DJ-1 mutant DA neurons [42]. Attenuation of this process results in the buildup of cargo proteins, which can trigger ER stress and activate the UPR response system, and prolonged ER stress caused by misfolded proteins has been linked to ROS production, possibly due to higher levels of protein folding activity in the ER lumen. Degradation of these misfolded proteins mitigates oxidative stress and associated cell death [96].

### Elevated intracellular DA promotes oxidation and increases SN DA neurons’ vulnerability towards oxidative stress

It is important to highlight that despite their relevance in PD phenotypes, α-synuclein, PINK1, Parkin, and DJ-1, are not exclusively expressed in midbrain DA neurons [3, 4, 6, 7, 36]. The specific neurodegeneration in loss of function mutants may be caused by the nature of the neurons themselves in their ability to produce and release DA, which can be highly reactive. Its metabolism can lead to the production of ROS byproducts such as hydrogen peroxide. DA synthesis involves several enzymatic reactions beginning with tyrosine hydroxylase catalyzing the hydroxylation of L-tyrosine at the phenol ring to produce L-3,4-dihydroxyphenylalanine (L-DOPA). Next, aromatic acid decarboxylase converts L-DOPA to DA. Active transport by vesicular monoamine transporter 2 (VMAT2) mediates DA storage in vesicles, an important step in protecting DA which is easily oxidized in the cytoplasm. In action potentials, DA vesicles fuse with the presynaptic membrane at the terminal button and are released into the synapse where they engage with receptors in the post-synaptic membranes. After binding, DA molecules can be reabsorbed into the cytosol via the DA transporter where it may undergo several different fates. It can be transported back into storage vesicles by VMAT2 and reused in the next axon firing, or be degraded by MAO, which leads to the production of 3,4-dihydroxyphenylacetaldehyde (DOPAL) and H_2_O_2_, two potent oxidizing agents [100, 101]. The enzyme aldehyde dehydrogenase turns DOPAL into the less reactive 3,4-dihydroxyphenylacetic acid (DOPAC) [101].

In the presence of iron in the cytosol, DA can be oxidized into DA-quinone (DAQ)—a highly reactive and toxic compound [102, 103]. In fact, the presence of the oxidized form of DA has been verified in iPSC-derived DA neurons containing *parkin, PINK1, LRRK2* mutations or *SNCA* triplication [42]. DAQ binds covalently with free cysteine and cysteine residues on proteins, which can drastically alter their function [104, 105]. It may also be involved in mitochondrial depolarization after DA exposure [106] and its binding to mitochondrial proteins can inhibit Complex I and IV. This was supported by the fact that treatment with quinone scavenger, GSH, reversed this effect. Proteomic analysis of isolated rat mitochondria in the same experiment revealed that mitochondrial proteins had been modified covalently by DAQ, including ubiquinol-cytochrome c reductase core protein 1 [107]. A recent study by Bondi et al. using SH-S5Y5 cells interestingly showed that DA treatment did not induce PINK and Parkin localization in the mitochondria, as CCCP treatment did. This means that despite inducing depolarization, DA did not activate the PINK1-Parkin autophagy pathway that was necessary to get rid of defective mitochondria [108]. In DJ-1 mutant human DA neurons, increased mitochondrial oxidative stress and accumulation of α-synuclein could be reversed with α-methyl-p-tyrosine, a competitive inhibitor of TH, preventing DA synthesis [42]. Accumulation of defective mitochondria by DA modification resembling mitochondria-related abnormalities were observed in several in vitro PD modeling studies involving PINK1 or Parkin mutations thus providing a basis for what may occur in sporadic cases and how the phenotypes mimic those of familial PD cases [25, 39, 40]. Moreover, a more oxidative environment due to defective mitochondria can further stimulate DA oxidation to DAQ, as indicated by increased binding of DAQ to cysteine-containing proteins in the striatum of animal models upon MPTP treatment [104]. The data altogether propose an alternate mechanism involving a positive feedback loop among PD elements that conditions the neurons into a state of chronic oxidative stress.

DA has also been shown to react and alter the function of PD related proteins, such as DJ-1, Parkin and α-synuclein [106, 109, 110]. In DA neurons, DA modification on Parkin leads to decreased solubility, functional inactivation, and subsequent accumulation of its ubiquitin ligase E3 substrates, including Synphilin-1 and Parkin, itself [111]. Interestingly, catechol-modified Parkin was only found in the SN of normal human brain tissue, but not in other areas such as caudate-putamen, cerebellum, and adjacent red nucleus [110]. Overexpression of α-synuclein in human primary DA neurons resulted in degeneration, a phenotype that was not observed in non-DA cells nor β-synuclein-overexpressing cells. Inhibition of DA production by α-methyl-p-tyrosine, a TH inhibitor, or antioxidant vitamin E reversed the α-synuclein overexpression-induced damage, supporting the hypothesis that DA fueled-oxidative stress plays a key role in mediating α-synuclein toxicity [112]. Furthermore, another study revealed that DOPAL-induced α-synuclein oligomers damaged cellular vesicles by permeabilizing cholesterol-containing vesicular membranes and inducing leakage of DA from vesicles into the cytosol [113]. DAQ non-covalent modification on α-synuclein was also observed to stabilize the protofibril form [114].

**Fig. 2:**
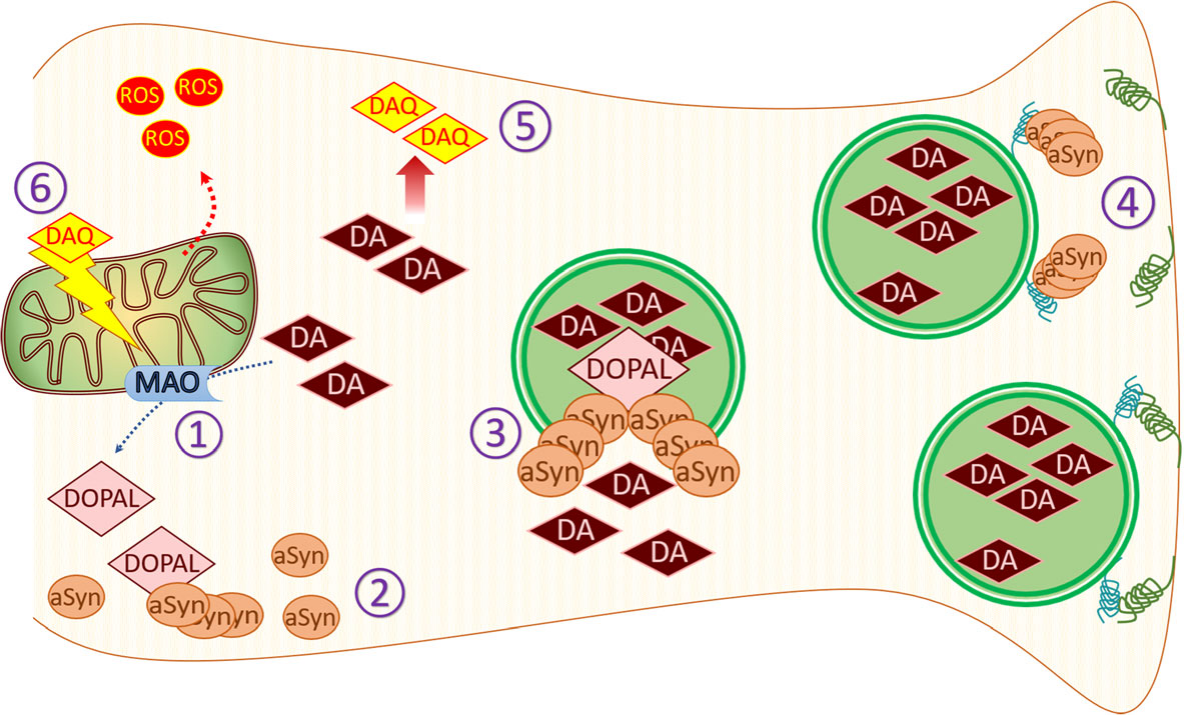
Alpha-synuclein oligomers and cytosolic DA amplify each other and synergistically contribute to oxidative stress. 1) DA anabolism enzyme, MAO, turns DA into DOPAL, that later can be converted into less reactive DOPAC by another enzyme. 2) DOPAL induces α-synuclein oligomerization and prevents fibril formation. 3) DOPAL modified α-synuclein oligomers create pore-like structure in the synaptic vesicle membrane, causing DA leakage into cytosol. 4) α-synuclein oligomers are also known to negatively regulate synaptic vesicle release by preventing SNARE formation and vesicle docking. This provides more DA vesicles that can be targeted by DOPAL-α-synuclein oligomers. 5) Oxidation of cytosolic DA produces DAQ. 6) DAQ reacts with cysteine residues of mitochondrial proteins that results in mitochondria depolarization and further ROS production

Changes in the level of cytosolic DA during PD progression remains a controversial subject, with studies arguing for elevation or [25, 115], reduction [26] as a cause of PD-related phenotypes. This contrast may be due to the decline in cytosolic DA, a feature of PD in its later stages when neurons are no longer able to produce DA in contrast to the earlier stages when overproduction of DA may be triggered by cellular dysfunction. The idea of DA overproduction is also supported by studies reporting the role of α-synuclein in negatively regulating DA vesicle release. It was found that a 2–3-fold α-synuclein overexpression in hippocampal and ventral midbrain neurons impeded synaptic vesicle release [116] while α-synuclein knock-out mice displayed stronger release of DA upon stimulus [117]. Two potential reasons behind this could be a reduction in the number of vesicles available for release [113, 116] or inhibition of vesicle docking by α-synuclein oligomers [118]. A follow-up study demonstrated that α-synuclein oligomers prevented soluble N-ethylmaleimide-sensitive factor attachment protein receptor (SNARE) complex formation which is necessary for vesicle docking, by binding to synaptobrevin-2, a vesicle-associated membrane protein [118]. These studies collectively demonstrate synergism between α-synuclein and DA in promoting oxidative stress in DA neurons (Fig. 2).

## Conclusion

The mechanism of neurodegeneration in PD still remains a controversial subject. Although PD entails a wide variety of cellular phenotypes, it is possible to decipher the events involved in the disease trajectory by studying the genetic forms of PD with the hopes of extrapolating gained insights toward the sporadic, non-familial forms. Mutations in PINK1, Parkin, DJ-1, and LRRK2 result in mitochondrial perturbations and elevations in oxidative stress. Utilization of DA as a neurotransmitter renders midbrain DA neurons more prone to damage given the potential for production of oxidative and reactive byproducts in DA metabolism. Elevated ROS levels also seem to be involved in metal-exposure related toxicity suspected to cause sporadic PD. These studies hint at the idea that oxidative stress plays a central role across a variety of PD linked phenotypes. Additionally, chronic oxidative stress, beyond the load that can be adequately regulated by homeostasis, can impact macromolecules inside the cell and result in cell death [20, 119].

Considering the complexity and singularity of each PD case, a great deal of effort is required to understand general PD pathology. Collecting PD case studies is an essential step in gaining a better idea of the underlying biology and overall landscape of the disease progression. Biological events considered in this review cannot explain the full spectrum of phenotypes present in PD like pathological events that occur on an intercellular level such as neuroinflammation [120], gut microbiota homeostasis [121], or the prion-hypothesis of α-synuclein [122]; however, mounting evidence pointing to oxidative stress as a common denominator provides hope for developing a more thorough understanding that could explain the complex cellular pathologies. This review proposes intracellular oxidative stress mitigation as major path toward regenerative treatment. Treatment with antioxidants, identification of appropriate antioxidant therapeutic candidates as well as efficient delivery methods across the blood brain barrier are major hurdles that would need to be resolved for building the groundwork for PD treatment in the context of our proposed paradigm. An additional implication is the identification of elements linked to oxidative stress as potential diagnostic targets for PD, including upregulation of lipid hydroperoxide and SOD activity, and downregulation of antioxidant factors like sulfhydryl groups and catalase activity in the blood [123]. Advances in the area of in vitro disease modeling have illuminated novel insights regarding PD and yielded new ways of studying complex cellular phenotypes. Despite these advantages, current in vitro or even animal disease models are limited by their inability to recapitulate the disease in an aged condition, which is especially relevant to studying neurodegenerative diseases which are chronic conditions that occur late in life. This issue remains a critical challenge given that there are no definitive methods of closely mimicking the naturally aged state of cells; nevertheless, given the rapid and continued progress in the field of disease modeling particularly in the context of neurological disorders, there is hope that these strides can soon lead to the development of effective therapeutic strategies.

## Abbreviations

6-OHDA: 6-hydroxydopamine
ATF: Activation transcription factor
CCCP: Carbonyl cyanide-m-chlorophenylhydrazone
CHOP: C/EBP homologous protein
DA: Dopamine
DAQ: DA-quinone
DOPAC: 3,4-dihydroxyphenylacetic acid
DOPAL: 3,4-dihydroxyphenylacetaldehyde
ER: Endoplasmic reticulum
GSH: Glutathione
iPSC: Induced pluripotent stem cell
IRE1: Inositol-requiring enzyme 1
L-DOPA: L-3,4-dihydroxyphenylalanine
LRRK2: Leucine-rich repeat kinase 2
MAM: Mitochondrial-associated membrane
MAO-B: Monoamine oxidase B
MCU: Mitochondrial calcium uniporter
MEF2C: Myocyte-specific enhancer factor 2C
MPP +: 1-methyl-4-phenylpyridinium
MPTP: 1-methyl-4-phenyl-1,2,3,6-tetrabydropyridine
mtDNA: Mitochondrial DNA
NADH: Nicotinamide adenine dinucleotide
PERK: Pancreatic ER kinase-like ER kinase
PD: Parkinson’s disease
PGC-1α: Peroxisome proliferator-activated receptor gamma coactivator 1-alpha
PINK1: PTEN induced putative kinase 1
ROS: Reactive oxygen species
SN: Substantia nigra
SNARE: Soluble N-ethylmaleimide-sensitive factor attachment protein receptor
SOD: Superoxide dismutase
TH: Tyrosine hydroxylase
TOM: Translocase of the outer membrane
UPR: Unfolded protein response
UPS: Ubiquitin-proteasome system
VMAT2: Vesicular monoamine transporter 2
VTA: Ventral tegmental area
WT: Wild type
XBP1: X-box binding protein 1
